# The effects of internet self-health management on patients with chronic disease multimorbidity: a 4-year longitudinal study

**DOI:** 10.3389/fdgth.2025.1568743

**Published:** 2025-07-30

**Authors:** Yuyang Wang, Qiang Hu, Botian Chen, Defu Ma

**Affiliations:** Department of Social Medicine and Health Education, School of Public Health, Peking University Health Science Center, Beijing, China

**Keywords:** multimorbidity, self-health management, internet health management, longitudinal study, mobile health

## Abstract

**Background:**

The escalating global burden of chronic diseases has given rise to a growing population affected by multimorbidity, defined as the co-occurrence of two or more chronic conditions. This health phenomenon is exacerbating disease burden through compounded clinical complications and increased healthcare demands. This study evaluates the effectiveness of internet-based self-health management in improving health behaviors and clinical indicators in patients with multimorbidity.

**Methods:**

A total of 30,745 adults aged ≥18 years from five northwestern Chinese provinces were enrolled. Following baseline data collection in 2013, participants received structured online health guidance covering diet nutrition, physical activity, and mental well-being. A follow-up assessment was conducted in 2017, involving questionnaire surveys and clinical measurements. Changes in health behaviors and clinical indicators of 2,535 patients with multimorbidity were analyzed. Binary logistic regression models were employed to identify factors influencing multimorbidity management outcomes.

**Results:**

The prevalence of multimorbidity at baseline in this study was 7.9%. After four years of health management, significant improvements were observed: smoking cessation rates increased from 8.2% to 10.2%, while low physical activity decreased from 29.0% to 24.6%. Both healthy individuals and multimorbid patients showed an increase in soybeans and nuts intake from 2013 to 2017. The fasting plasma glucose of the multimorbidity subjects decreased from 9.33 mmol/L in 2013 to 8.28 mmol/L in 2017, and the total cholesterol level decreased from 6.97 mmol/L to 6.26 mmol/L (*P* < 0.001). Significant reductions were also observed in triglycerides and low-density lipoprotein cholesterol levels (*P* < 0.001). The binary logistic regression results showed that being 40 years or older, male, having a family history of chronic diseases, changes in smoking status and sleep quality under health management guidance were influencing factors for effective control of multimorbidity.

**Conclusion:**

Internet-based self-health management effectively improves health behaviors and clinical indicators in patients with chronic disease multimorbidity.

## Highlights

• This study assesses the effect of internet-based health management on the clinical parameters and health behaviors of patients with multimorbidity.
• Internet-based self-health management significantly improves health behaviors and clinical indicators in patients with multimorbidity over a four-year period.
• Key findings include increased smoking cessation rates, reduced physical inactivity, and improved clinical parameters such as fasting plasma glucose and cholesterol levels in multimorbid patients.
• This study identifies age, gender, family history of chronic diseases, and changes in smoking and sleep behaviors as critical factors influencing the effective control of multimorbidity.

## 1 Introduction

The global epidemiological landscape has undergone significant transformations due to shifting lifestyle patterns, population aging, and increased life expectancy, leading to chronic diseases emerging as the predominant threat to human health. Characterized by complex etiologies and prolonged disease courses, chronic conditions frequently co-occur, resulting in disease accumulation (1). The World Health Organization (WHO) formally recognized this phenomenon in 2008 by introducing the concept of multimorbidity—the coexistence of two or more chronic conditions in an individual (2). Current epidemiological data reveal the substantial global burden of multimorbidity. Approximately one-third of adults worldwide, including significant proportions in low- and middle-income countries, are affected (3). Regional studies demonstrate prevalence rates of 27.2% in U.S. adults (4), 39.6% in Canadian adults (with an average of 2.41 chronic conditions per affected individual) (5), and nearly 25% in Chinese adults, rising to 42.4% among elderly populations (6, 7). The clinical and public health implications of multimorbidity are profound. Each additional chronic condition is associated with 3.2 extra hospital visits annually and a 36% increased mortality risk (8–10). Compared to single-disease patients, those with multimorbidity experience significantly worse health outcomes, greater treatment complexity, and substantial challenges in disease management, making it one of the most pressing challenges facing healthcare systems worldwide.

The treatment of patients with multimorbidity relies solely on the medical support of health care professionals, which is far from sufficient. It necessitates continuous self-health management, which includes consistent monitoring of symptoms, adherence to treatment plans, and lifestyle adjustments to control the onset and progression of diseases. Previous studies have found that proactive health management can effectively improve the health behaviors, health status, and utilization of health services among patients with chronic diseases (11–13). Nevertheless, the implementation of sustained and personalized health management has become a major challenge for patients with chronic diseases.

With the continuous advancement of technology, the role of internet-based health management in the management of chronic diseases is becoming increasingly significant. Internet-based health management services offer a convenient tool to enhance patient compliance and accessibility, potentially improving treatment outcomes, reducing costs, and enhancing overall quality of life (14–17). To date, studies reporting on internet-based health management have primarily been limited to patients with single chronic diseases, including obesity (18), hypertension (19), dyslipidemia (20), and diabetes (21). However, evidence regarding the effectiveness of internet-based health management in managing multimorbidity remains limited.

Therefore, this study assesses the long-term impact of internet-based health management on the clinical parameters of patients with multimorbidity. Moreover, the study seeks to elucidate the factors influencing the health status of patients with multimorbidity, offering a scientific rationale for the adoption of personalized health management strategies to improve the quality of life for patients with multimorbidity.

## 2 Methods

### 2.1 Study population

A longitudinal research method is performed to assess the effects of implementing internet-based health management on patients with multimorbidity. The study is based on the employee health promotion program carried out by a large Chinese petroleum enterprise. The overall objective of this program is to assess the intervention effects of internet-based health management on the health status and lifestyle of the working population.

The participants of this study were recruited from the enterprise in five provinces of China, namely Shaanxi, Gansu, Ningxia, Inner Mongolia, and Shanxi, from January to May 2013. After the enterprise held project briefing sessions, demonstrated the operation of the internet health platform, and explained the screening criteria in detail, the branches of the enterprise organized volunteers to sign up voluntarily. The inclusion criteria for the study subjects were: (1) age ≥18 years; (2) proficient in using a smartphone; (3) willingness to cooperate with the study and participate in regular follow-ups. Patients with severe illnesses were excluded, as they typically require intensive and specialized medical care, and their complex conditions might interfere with the assessment of intervention outcomes. Volunteers were enrolled through the enterprise's branch offices, resulting in a total of 56,542 individuals from 15 cities and 61 counties (districts and banners) joining the internet health management platform.

After the baseline survey, participants received health management guidance and follow-ups through the internet health management platform and underwent another round of physical examinations and questionnaires in 2017. Ultimately, complete baseline information was obtained from 51,486 individuals, among whom 30,745 participants took part in the follow-up survey in 2017 and reported complete information. All participants in the study signed informed consent forms and voluntarily joined the study. This study adhered to the guidelines of the Declaration of Helsinki (1975) and was reviewed and approved by the Peking University Biomedical Ethics Committee (IRB00001052-0816).

### 2.2 Internet-based health management platform

Each participant registers and creates a personal account on the health management platform, which is exclusively for their use. The platform is divided into three modules: “Understanding my health”, “Improving my health”, and “My health management outcomes”. After uploading the baseline physical examination report to the platform, a personal health record is established. Users can access their health records anytime in the first module to review their current health status and identify existing health issues through the platform's assessment. In addition to basic information (e.g., age, height, weight, waist circumference), the health record includes clinical indicators such as lipid profiles, blood glucose, blood pressure, personal and family medical histories, and evaluations of lifestyle behaviors (e.g., smoking status, alcohol consumption, dietary habits, physical activity levels). Following the assessment, the platform formulates a personalized health management plan in the second module based on the user's health status information. This plan includes tailored dietary, exercise, and psychological adjustment programs, as well as recommendations for mitigating other identified health risk behaviors. For smoking behavior, the system will push evidence-based phased smoking cessation strategies, track changes in smoking behavior through questionnaires, and continuously provide encouragement messages or alternative behavior suggestions to the participants through the health management platform. For sleep management, the platform combines with the mental health guidance module to provide sleep hygiene education (such as suggestions for regular work and rest schedules), relaxation training resources (such as mindfulness meditation audio), and a sleep quality tracking function. Users can manually record their sleep duration and quality to obtain personalized adjustment suggestions. In addition, the platform will regularly push popular science articles and videos related to blood pressure, blood lipid, blood glucose, weight management, smoking cessation techniques, and sleep improvement methods. The content is reviewed by public health experts to ensure its scientificity and practicality. After three months of health management, the third module further evaluates the effectiveness of the initial management interventions.

Users must log in to the platform at least once a week to update data, including dietary records and exercise duration. When they log in, the system will push weekly dietary and exercise plans, along with health education tasks. Additionally, users need to complete at least one full record within 5 days, and it is advisable that each interaction lasts between 20 and 30 min. In order to balance the needs of structuring and personalization, the platform allows users to adjust the intensity of their participation according to their own situations. Throughout the entire process of Internet-based health management, healthcare professionals mainly provide remote personalized support via phone calls or text messages. In addition, the platform also tracks the daily login status and usage patterns of the participants. If a participant has not used the platform for more than a week, the researchers will contact them by phone and record the reasons for their inactivity.

### 2.3 Dietary management

After completing the questionnaire, the platform automatically generates a dietary prescription based on the survey results. Participants can select and substitute foods from the menus recommended by nutrition experts. Users are required to record at least two days of dietary intake per week on the platform. The system analyzes their diet and nutritional status, identifies existing dietary issues, and provides targeted recommendations.

### 2.4 Exercise management

The health management platform automatically formulates a 12-phase progressive exercise prescription based on the user's health status. Participants can choose recommended exercise activities, including aerobic exercises, strength training, and flexibility exercises, and manually record their exercise dates and durations. The system automatically calculates energy expenditure post-exercise. Each week, the system evaluates the current exercise prescription's appropriateness by comparing the previous week's energy expenditure with the recommended exercise prescription.

### 2.5 Definition of multimorbidity

In this study, the chronic disease status of each participant was confirmed through self-reported physician diagnosis or in combination with clinical testing. Referring to previous studies and taking into account the significant public health burden and clinical relevance, the chronic diseases involved in this study include hypertension, diabetes, dyslipidemia, heart disease, stroke, chronic bronchitis, asthma, emphysema, tuberculosis, lung cancer, and chronic obstructive pulmonary disease (COPD) (22). These diseases not only have a high prevalence in the population but also often interact with each other due to the intertwining of pathological mechanisms. Multimorbidity was defined as the presence of two or more of these chronic diseases within the same individual. Multimorbidity control is defined as the reduction in the number of chronic diseases listed above to one or fewer in a multimorbid patient following management and treatment.

### 2.6 Clinical parameter measurement

After an overnight fast of at least 8 h, fasting venous blood was collected from the subjects without the use of anticoagulants. Fasting plasma glucose (FPG) was measured using the glucose oxidase method, and serum lipid levels, including total cholesterol (TC), triglycerides (TG), high-density lipoprotein cholesterol (HDL-C), and low-density lipoprotein cholesterol (LDL-C), were determined enzymatically. All these measurements were performed using a fully automated biochemical analyzer. Blood pressure was measured using an electronic sphygmomanometer. Participants were required to sit quietly for at least 5 min to achieve a relaxed state before the measurements. Blood pressure was measured twice, with an interval of more than 30 s between measurements. The average of the two consecutive measurements was used for analysis.

### 2.7 Data collection

After each physical examination, demographic information, lifestyle behaviors (smoking status, alcohol consumption, dietary habits, physical activity levels, sleep patterns), and personal and family medical histories were collected via questionnaires. Physical activity was categorized into low, moderate, and high levels according to the standards provided by the International Physical Activity Questionnaire (IPAQ) (23). Dietary intake was scored based on the 2016 Chinese Dietary Guidelines, classifying it as below recommended intake, recommended intake, and above recommended intake (24). The General Health Questionnaire (GHQ-12) was used to assess the psychological status of the subjects (25). Improvements in the results from 2017 compared to those from 2013 were considered indicative of reduced risk behaviors following the health management program.

### 2.8 Statistical analysis

The original data were processed using the 99th percentile truncation method to eliminate extreme outliers. For the description of the basic characteristics of the study subjects, continuous variables presented as mean ± standard deviation (SD), and categorical variables reported as percentages(%). The Chi-square test was used to compare the baseline characteristics of different populations with chronic diseases. Student's *t*-tests and Chi-square tests were employed to analyze changes in clinical indicators, lifestyle habits, and dietary patterns of the study subjects through internet health management. Patients with multimorbidity were grouped based on their disease control status after four years of health management, and the Chi-square test was used to analyze factors that might influence the control of multiple conditions. These factors were further analyzed using a binary logistic regression model, expressed as odds ratios (OR) and 95% confidence intervals (CI). All statistical analyses were performed using SPSS 21.0 software, with all *P*-values subjected to two-tailed tests at a significance level of 0.05.

## 3 Results

### 3.1 Basic characteristics of the population

The 2013 baseline survey enrolled 56,542 participants (62.6% male, 37.4% female). Among the baseline population, 36,466 individuals (64.5%) had no chronic diseases, 15,614 individuals (27.6%) had one chronic disease, and 4,482 individuals (7.9%) suffered from multimorbidity, defined as having two or more chronic diseases simultaneously. Except for marital status, education level, intake of fish, livestock meat, eggs, and vegetables, the differences in other baseline characteristics (age, gender, smoking, alcohol consumption, cereals and potato intake, physical activity, etc.) between the three groups were statistically significant. Regardless of the group, the intake of milk, fruits, and vegetables among the study subjects was inadequate (Table 1).

**Table 1:** Basic characteristics of all participants in 2013.

Characteristics	Total (*n* = 56,542)	Number of Chronic Conditions			*P*
		None (*n* = 36,446)	One (*n* = 15,614)	Two or More (*n* = 4,482)	
Age (mean ± SD)	36.8 ± 9.0	35.8 ± 8.3	37.4 ± 9.6	40.9 ± 9.8	<0.001
Gender, *n* (%)					<0.001
Male	35,412 (62.6)	20,204 (55.4)	11,592 (74.2)	3,616 (80.7)	
Female	21,130 (37.4)	16,242 (44.6)	4,022 (25.8)	866 (19.3)	
Marital status, *n* (%)					0.74
Married	48,021 (84.9)	30,900 (84.8)	13,294 (85.1)	3,827 (85.4)	
Unmarried	6,794 (12.0)	4,421 (12.1)	1,848 (11.8)	525 (11.7)	
Divorced	1,727 (3.1)	1,125 (3.1)	472 (3.0)	130 (2.9)	
Education level, *n* (%)					0.23
Junior/Middle School	12,951 (22.9)	8,381 (23.0)	3,605 (23.1)	965 (21.5)	
Senior High School	9,681 (17.1)	6,222 (17.1)	2,665 (17.1)	794 (17.7)	
College and above	33,910 (60.0)	21,843 (59.9)	9,344 (59.8)	2,723 (60.8)	
Family history of chronic diseases, *n* (%)					<0.001
Yes	11,748 (20.8)	6,390 (17.5)	3,521 (22.6)	1,837 (41.0)	
No	44,794 (79.2)	30,056 (82.5)	12,093 (77.4)	2,645 (59.0)	
Smoking, *n* (%)					<0.001
Never smoked	36,833 (65.2)	25,772 (70.8)	9,010 (57.7)	2,051 (45.8)	
Smoking	18,065 (32.0)	9,969 (27.4)	6,011 (38.5)	2,085 (46.5)	
Quit smoking	1,606 (2.8)	676 (1.9)	584 (3.7)	346 (7.7)	
Drinking, *n* (%)					<0.001
Never drank	34,044 (66.1)	23,229 (70.4)	8,498 (60.1)	2,317 (52.8)	
Drinking	17,473 (33.9)	9,757 (29.6)	5,646 (39.9)	2,070 (47.2)	
Physical activity, *n* (%)					<0.001
Low	17,316 (30.6)	11,013 (30.2)	4,932 (31.6)	1,371 (30.6)	
Moderate	26,126 (46.2)	17,062 (46.8)	7,125 (45.6)	1,939 (43.3)	
High	13,100 (23.2)	8,371 (23.0)	3,557 (22.8)	1,172 (26.1)	
Quality of sleep, *n* (%)					<0.001
Very good	12,939 (23.0)	8,375 (23.1)	3,817 (24.6)	747 (16.7)	
Fair	34,877 (62.0)	22,957 (63.3)	9,337 (60.2)	2,583 (57.7)	
Not good	6,957 (12.4)	4,115 (11.4)	1,935 (12.5)	907 (20.3)	
Very bad	1,469 (2.6)	794 (2.2)	433 (2.8)	242 (5.4)	
Cereals and potato intake, *n* (%)					<0.001
Below	24,342 (43.1)	15,981 (43.8)	6,638 (42.5)	1,723 (38.4)	
Moderate	16,798 (29.7)	11,112 (30.5)	4,471 (28.6)	1,215 (27.1)	
Higher	15,402 (27.2)	9,353 (25.7)	4,505 (28.9)	1,544 (34.4)	
Fish, livestock meat, and eggs intake, *n* (%)					0.06
Below	29,614 (52.4)	19,100 (52.4)	8,246 (52.8)	2,268 (50.6)	
Moderate	13,654 (24.1)	8,839 (24.3)	3,682 (23.6)	1,133 (25.3)	
Higher	13,274 (23.5)	8,507 (23.3)	3,686 (23.6)	1,081 (24.1)	
Milk and dairy products intake, *n* (%)					<0.001
Below	50,213 (88.8)	32,187 (88.3)	13,991 (89.6)	4,035 (90.0)	
Moderate or higher	6,329 (11.2)	4,259 (11.7)	1,623 (10.4)	447 (10.0)	
Soybeans and nuts intake, *n* (%)					<0.001
Below	37,671 (66.6)	23,921 (65.6)	10,654 (68.2)	3,096 (69.1)	
Moderate	3,112 (5.5)	2,175 (6.0)	716 (4.6)	221 (4.9)	
Higher	15,759 (27.9)	10,350 (28.4)	4,244 (27.2)	1,165 (26.0)	
Vegetables intake, *n* (%)					0.51
Below	42,821 (75.7)	27,550 (75.6)	11,859 (76.0)	3,412 (76.1)	
Moderate	9,291 (16.4)	5,986 (16.4)	2,572 (16.5)	733 (16.4)	
Higher	4,430 (7.8)	2,910 (8.0)	1,183 (7.6)	337 (7.5)	
Fruit intake, *n* (%)					<0.001
Below	48,984 (86.6)	31,227 (85.7)	13,721 (87.9)	4,036 (90.0)	
Moderate	5,702 (10.1)	3,926 (10.8)	1,448 (9.3)	328 (7.3)	
Higher	1,856 (3.3)	1,293 (3.5)	445 (2.9)	118 (2.6)	

Continuous variables are presented as mean ± SD; categorical variables are shown as percentages (*%*).

### 3.2 Changes in lifestyle and dietary intake after four years of self-health management

Among the 30,745 participants who completed follow-up assessments (including 26,028 healthy controls and 2,535 multimorbidity cases), four years of internet-based self-health management resulted in significant behavioral changes. Smoking cessation rates increased from 2.5% to 4.2% in healthy participants and from 8.2% to 10.2% in the multimorbidity group. Alcohol consumption rates increased among healthy participants from 33.2% to 37.3%, while the proportion with low physical activity decreased from 28.8% to 24.9%. Both groups showed increased consumption of soybeans and nuts, with healthy participants also increasing fruit intake. The multimorbidity group demonstrated reduced physical inactivity (29.0% to 24.6%) (Table 2).

**Table 2:** Changes in lifestyle and dietary intake after four years of self-health management.

Characteristics	Healthy (*n* = 26,028)		*P*	Multimorbidity (*n* = 2,535)		*P*
	2013	2017		2013	2017	
Smoking, *n* (%)			<0.001			0.04
Never smoked	16,713 (64.2)	15,986 (61.4)		1,118 (44.1)	1,077 (42.5)	
Smoking	8,653 (33.2)	8,942 (34.4)		1,208 (47.7)	1,199 (47.3)	
Quit smoking	662 (2.5)	1,100 (4.2)		209 (8.2)	259 (10.2)	
Drinking, *n* (%)			<0.001			0.10
Never drank	17,386 (66.8)	16,322 (62.7)		1,325 (52.9)	1,283 (50.6)	
Drinking	8,642 (33.2)	9,706 (37.3)		1,180 (47.1)	1,252 (49.4)	
Physical activity, *n* (%)			<0.001			<0.001
Low	7,490 (28.8)	5,719 (24.9)		734 (29.0)	547 (24.6)	
Moderate	11,859 (45.6)	11,929 (51.8)		1,137 (44.9)	1,139 (51.3)	
High	6,679 (25.7)	5,361 (23.3)		664 (26.2)	536 (24.1)	
Quality of sleep, *n* (%)			<0.001			0.51
Very good	5,923 (22.8)	5,011 (19.3)		411 (16.2)	375 (14.8)	
Fair	15,931 (61.2)	15,772 (60.6)		1,425 (56.2)	1,430 (56.4)	
Not good	3,447 (13.2)	4,275 (16.4)		540 (21.3)	565 (22.3)	
Very bad	718 (2.8)	970 (3.7)		159 (6.3)	165 (6.5)	
Cereals and potato intake, *n* (%)			0.62			0.65
Below	11,138 (42.8)	11,087 (42.6)		962 (37.9)	936 (36.9)	
Moderate	7,558 (29.0)	7,659 (29.4)		716 (28.2)	743 (29.3)	
Higher	7,332 (28.2)	7,282 (28.0)		857 (33.8)	856 (33.8)	
Fish, livestock meat, and eggs intake, *n* (%)			0.13			0.65
Below	12,821 (49.3)	12,591 (48.4)		1,298 (51.2)	1,267 (50.0)	
Moderate	6,579 (25.3)	6,685 (25.7)		646 (25.5)	654 (25.8)	
Higher	6,628 (25.5)	6,752 (25.9)		591 (23.3)	614 (24.2)	
Milk and dairy products intake, *n* (%)			0.70			0.93
Below	23,021 (88.4)	23,049 (88.6)		2,276 (89.8)	2,278 (89.9)	
Moderate or higher	3,007 (11.6)	2,979 (11.4)		259 (10.2)	257 (10.1)	
Soybeans and nuts intake, *n* (%)			<0.001			<0.001
Below	16,992 (65.3)	11,895 (45.7)		1,751 (69.1)	1,218 (48.0)	
Moderate	1,567 (6.0)	3,029 (11.6)		117 (4.6)	310 (12.2)	
Higher	7,469 (28.7)	11,104 (42.7)		667 (26.3)	1,007 (39.7)	
Vegetables intake, *n* (%)			0.49			0.42
Below	19,403 (74.5)	19,310 (74.2)		1,932 (76.2)	1,892 (74.6)	
Moderate	4,530 (17.4)	4,553 (17.5)		411 (16.2)	441 (17.4)	
Higher	2,095 (8.0)	2,165 (8.3)		192 (7.6)	202 (8.0)	
Fruit intake, *n* (%)			0.03			0.18
Below	22,485 (86.4)	22,352 (85.9)		2,286 (90.2)	2,259 (89.1)	
Moderate	2,620 (10.1)	2,639 (10.1)		189 (7.5)	195 (7.7)	
Higher	923 (3.5)	1,037 (4.0)		60 (2.4)	81 (3.2)	

### 3.3 The effectiveness of clinical parameter control before and after self-health management

After four years of self-health management, healthy study subjects had significantly higher SBP, FPG, TG, LDL-C, and BMI, and significantly lower HDL-C levels (*P* < 0.05). The FPG of the study subjects with multimorbidity decreased from 9.33 mmol/L in 2013 to 8.28 mmol/L in 2017, TC levels decreased from 6.97 mmol/L to 6.26 mmol/L, and significant decreases in TG and HDL-C levels also occurred (*P* < 0.001). Decreases in LDL-C and BMI levels in the multimorbidity population also occurred from 2013 to 2017, respectively, although the differences were not statistically significant (Table 3).

**Table 3:** Changes in clinical parameters after four years of self-health management.

Clinical parameters	Healthy (*n* = 26,028)		*P*	Multimorbidity (*n* = 2,535)		*P*
	2013	2017		2013	2017	
SBP (mmHg)	117.15 ± 13.43	117.58 ± 13.83	<0.001	127.54 ± 17.85	127.80 ± 17.35	0.59
DBP (mmHg)	76.29 ± 10.17	76.16 ± 10.43	0.16	84.39 ± 13.07	83.69 ± 12.66	0.05
FPG (mmol/L)	4.96 ± 2.00	5.00 ± 1.96	0.04	9.33 ± 5.78	8.28 ± 5.30	<0.001
TC (mmol/L)	4.42 ± 2.44	4.42 ± 2.39	0.81	6.97 ± 5.58	6.26 ± 4.91	<0.001
TG (mmol/L)	1.71 ± 1.49	1.76 ± 1.48	<0.001	4.17 ± 3.89	3.65 ± 3.49	<0.001
HDL-C (mmol/L)	1.86 ± 1.07	1.81 ± 1.04	<0.001	2.58 ± 2.72	2.29 ± 2.35	<0.001
LDL-C (mmol/L)	1.77 ± 0.92	1.85 ± 0.93	<0.001	2.46 ± 2.30	2.37 ± 1.99	0.13
BMI (kg/m^2^)	23.15 ± 3.78	23.25 ± 3.54	<0.001	25.04 ± 4.00	24.92 ± 3.67	0.26

SBP, systolic blood pressure; DBP, diastolic blood pressure; FPG, fasting plasma glucose; TC, total cholesterol; TG, triglycerides; HDL-C, high-density lipoprotein cholesterol; LDL-C, low-density lipoprotein cholesterol; BMI, body mass index.

### 3.4 Demographic and health behaviors related to the effective control of multimorbidity

Of the 2535 people with multimorbidity at the time of the baseline survey, 506 had effective controlled their conditions after four years of health management, while 2,029 did not, resulting in an effective control rate for multimorbidity of 19.96% (Table 4). Among patients under the age of 40, 26.6% achieved multimorbidity control. The control rate in males was 16.7% while in females it was higher at 34.0% (*P* < 0.001). Patients with no family history of chronic disease had a higher control rate of 23.7% (*P* < 0.001). In terms of lifestyle factors, patients who adhered to health management and improved their smoking behavior (either by quitting or reducing smoking) had an higher effective control rate of 25.3% (*P* < 0.001). Patients who reduced alcohol intake also had an effective control rate of 22.6%, which was higher than the 17.4% of those who did not make any changes (*P* < 0.001). In addition, improving sleep quality to better and above levels according to health management was effective in controlling multimorbidity (*P* < 0.05).

**Table 4:** Demographic and health behaviors related to the effective control of multimorbidity.

Factors	Effective multimorbidity control (*n* = 506)	Ineffective multimorbidity control (*n* = 2,029)	*P*
Demographic characteristics			
Age, *n* (%)			<0.001
<40 years	338 (26.6)	931 (73.4)	
≥40 years	168 (13.3)	1,098 (86.7)	
Gender, *n* (%)			<0.001
Male	344 (16.7)	1,714 (83.3)	
Female	162 (34.0)	315 (66.0)	
Family history of chronic diseases, *n* (%)			<0.001
Yes	253 (17.3)	1,213 (82.7)	
No	253 (23.7)	816 (76.3)	
Improved health behaviors following health management^a^			
Smoking, *n* (%)			<0.001
Yes	313 (25.3)	925 (74.7)	
No	193 (14.9)	1,104 (85.1)	
Drinking, *n* (%)			<0.001
Yes	284 (22.6)	975 (77.4)	
No	222 (17.4)	1,054 (82.6)	
Physical activity, *n* (%)			0.65
Yes	330 (19.7)	1,345 (80.3)	
No	176 (20.5)	684 (79.5)	
Sleeping quality, *n* (%)			0.04
Yes	379 (21.0)	1,426 (79.0)	
No	127 (17.4)	603 (82.6)	
Cereals and potato intake, *n* (%)			0.03
Yes	298 (18.6)	1,301 (81.4)	
No	208 (22.2)	728 (77.8)	
Fish, livestock meat, and eggs intake, *n* (%)			0.61
Yes	248 (19.6)	1,020 (80.4)	
No	258 (20.4)	1,009 (79.6)	
Milk and dairy products intake, *n* (%)			0.38
Yes	46 (17.9)	211 (82.1)	
No	460 (20.2)	1,818 (79.8)	
Soybeans and nuts intake, *n* (%)			0.42
Yes	271 (20.6)	1,046 (79.4)	
No	235 (19.3)	983 (80.7)	
Vegetables intake, *n* (%)			0.05
Yes	111 (17.3)	532 (82.7)	
No	395 (20.9)	1,497 (79.1)	
Fruit intake, *n* (%)			0.27
Yes	62 (22.5)	214 (77.5)	
No	444 (19.7)	1,815 (80.3)	

^a^ Changes in unhealthy lifestyles among study participants following health management guidance.

### 3.5 Factors affecting the control of multimorbidity after health management

The results of the binary logistic regression indicate that patients with multimorbidity ≥40 years of age have worse control outcomes after health management, with a risk of ineffective control that was 2.039 times (95% CI: 1.649–2.521) higher than that of participants <40 years old (Table 5). Male patients with multimorbidity have a higher risk of ineffective control compared to female patients, with an OR of 1.951 (95% CI: 1.500–2.536). Patients with a family history of chronic diseases have a higher risk of ineffective multimorbidity control. Additionally, regarding lifestyle factors, patients with multimorbidity who failed to adhere to health management to improve smoking behavior had a 1.313 times (95% CI: 1.038–1.661) higher risk of control failure compared to those who made changes, and those who failed to improve sleep quality had a 1.297 times (95% CI: 1.029–1.634) higher risk compared to those who made changes.

**Table 5:** Binary logistic regression analysis of factors affecting the control of multimorbidity after health management.

Variable	Estimate (B)	OR (95% CI)	*P*
Demographic factors			
Age			<0.001
<40years		1 (Ref.)	
≥40years	0.712	2.039 (1.649, 2.521)	
Gender			<0.001
Female		1 (Ref.)	
Male	0.668	1.951 (1.500, 2.536)	
Family history of chronic diseases			<0.001
No		1 (Ref.)	
Yes	0.314	1.369 (1.117, 1.679)	
Improved health behaviors following health management^a^			
Smoking			0.02
Yes		1 (Ref.)	
No	0.272	1.313 (1.038, 1.661)	
Sleeping quality			0.03
Yes		1 (Ref.)	
No	0.260	1.297 (1.029, 1.634)	
Constant	0.194	1.215	0.11

^a^ Changes in unhealthy lifestyles among study participants following health management guidance.

## 4 Discussion

This study assessed the control effects of chronic multimorbidity patients after four years of Internet-based health management. Internet-based health management implemented for patients with multimorbidity proved to be effective. Among them, 19.96% of the patients with multimorbidity were effectively controlled, which means that the number of chronic diseases involved in this study that they suffered from decreased from two or more to one or fewer. The results showed that the study population had improvements in smoking cessation rates, physical activity levels, soy and nut intake, and fruit intake occurring in the study population, and reductions in the levels of FPG, TC, LDL-C, and BMI indicators occurring in patients with multimorbidity. In addition, Furthermore, patients under 40 years old, females, and those who improved their smoking and sleep quality showed significant benefits in controlling multimorbidity when following health management guidance.

He et al. (26) reported a prevalence of multimorbidity among residents in mainland China from 1998 to 2019 of 36.3%, while the prevalence of multimorbidity in the baseline survey of the present study was 7.9%, which is closer to the reported rate of 10.6% for individuals under 40 years old in their report. This may be related to the relatively younger average age of the study population in the current research. After four years of internet-based self-health management, 19.96% of individuals with chronic diseases in this study achieved effective control of their multimorbid conditions. Xiao and colleagues (27) have confirmed the positive effects of a telemedicine chronic disease management system on the health and quality of life of patients with chronic diseases. Lorig and colleagues, through a randomized trial, found that patients with chronic diseases who engaged in internet-based self-management experienced significant improvements in their health status (17). Chen et al. discovered that internet-based health management can effectively reduce the risk factors for metabolic syndrome among professional women (28). Internet-based health management increases the accessibility of remote health services for individuals with chronic diseases, particularly benefiting those with limited mobility (29). By receiving convenient health management guidance, patients can start self-managing their lifestyle earlier, before severe outcomes occur, thereby reducing the risk of more serious disease events and potential economic losses (30).

This study found that patients with multimorbidity had the highest smoking rate, and after four years of internet-based health management, an increase in smoking cessation rates was observed in both the healthy population and those with multimorbidity. A cross-sectional study showed that the smoking rate among people with coronary heart disease and its multimorbidities was more than twice that of those without multimorbidity, and those with multimorbidity were less likely to follow smoking cessation advice (31). Wilson and colleagues found through a mobile health emergency management initiative conducted among American veterans that participants in the intervention group were more likely to achieve long-term smoking cessation (32). A study based on web-based personalized smoking cessation programs found that participants had high engagement, and the smoking cessation rate significantly increased among the Mexican population at the end of the intervention (33). In addition, this study found that reducing tobacco use under the guidance of health management was an effective factor in controlling the impact of multimorbidity. Tobacco use has been found to be associated with an increased risk of more than 27 diseases, therefore, patients with multiple diseases are more in need of smoking cessation than the average smoker (34, 35).

This study found that the physical activity levels of patients with multimorbidity improved after health management, with more patients transitioning from low to moderate or high levels of physical activity. Khunti and colleagues discovered through a randomized controlled trial that a self-management plan led to a slight decrease in the physical activity levels of patients with multiple diseases (36). Patients with multimorbidity often have lower levels of physical activity, which is related to various factors such as their physical capacity and mental health, therefore selecting an appropriate exercise management approach for patients with multimorbidity is more important when implementing health management (37). This study observed that improvements in sleep quality could effectively control multimorbidity, and a cross-sectional study from Luxembourg showed a linear association between shorter sleep duration and the number of chronic conditions (38). In the middle-aged and elderly population in China, poor sleep quality was associated with a higher risk of progression of multimorbidity (39). Sleep quality has a significant impact on metabolic functions, inflammatory responses, and endocrine functions; poor sleep quality can lead to increased levels of pro-inflammatory factors and cortisol, promoting stress and inflammatory responses, thus inhibiting effective control of the conditions in patients with multimorbidity (40).

This study reports a decrease in the levels of FPG, TC, TG, HDL-C, LDL-C, and BMI among patients with multimorbidity after internet-based health management. Li and colleagues found that self-health management conducted through mobile devices effectively reduced LDL-C levels in patients with atherosclerotic cardiovascular disease (41). A trial assessing self-health management in Americans with diabetes and hypertension found that the intervention group was more effective in controlling blood glucose, with lower levels of hemoglobin A1c (HbA1c) (42). Internet-based health management for patients with multimorbidity, which includes dietary and exercise guidance and intensive lifestyle interventions, improves metabolic function by affecting insulin sensitivity and glucose homeostasis (43, 44). Additionally, this study observed improvements in the intake of soy and nuts, as well as fruit consumption among the subjects. A reasonable diet helps patients with multimorbidity improve vascular endothelial function and reduce inflammation and oxidative stress. Lifestyle interventions based on health management may be an effective strategy for improving chronic inflammation in patients with multimorbidity (45).

This study has several limitations. Firstly, although the sample size of this study was large, the subjects were selected only from five provinces in northwestern China, and the results may not be generalizable to other regions due to differences in economic levels and other factors. Secondly, this study adopted a longitudinal research design. While the changes of various parameters over time were observed, due to the lack of a control group, social media, community health education and management, as well as other confounding factors, may affect the evaluation of the intervention effect. Moreover, this study was conducted among employees of large enterprises, and a relatively high dropout rate was caused by factors such as job changes or personnel adjustments. All these factors may limit the universality of the research results.

To address these issues, multi-center randomized controlled trials should be adopted in the future, and the health management platform should be further optimized to better improve the compliance of the participants and determine the independent effects of the intervention measures. In addition, although improvements in health behaviors and clinical indicators were observed over the 4 years period, this study did not assess whether these improvements persisted after the conclusion of the study. Therefore, the long-term effects and sustainability of internet-based health management require further investigation in the future.

## 5 Conclusions

This longitudinal study confirms that internet-based health management is effective in managing chronic disease multimorbidity. Through health management guidance, it is possible to achieve changes in the unhealthy lifestyles of patients with multimorbidity and observe significant changes in clinical indicators after four years. In the future, more internet health management strategies should be adopted to better prevent and control the occurrence and development of multimorbidity through this economical, convenient, and accessible approach.

## Data Availability

The raw data supporting the conclusions of this article will be made available by the authors, without undue reservation.
